# The cGMP signaling pathway affects feeding behavior in the necromenic nematode *Pristionchus pacificus*

**DOI:** 10.1186/1753-6561-6-S3-P27

**Published:** 2012-06-01

**Authors:** Silvina M Kroetz, Jagan Srinivasan, Jonathan Yaghoobian, Paul W Sternberg, Ray L Hong

**Affiliations:** 1 California State University Northridge, Department of Biology, Northridge, CA USA; 2 California Institute of Technology, Division of Biology, Pasadena, CA, USA

## Background

The genetic tractability and the species-specific association with beetles make the nematode *Pristionchus pacificus* an exciting emerging model organism for comparative studies in development and behavior. *P. pacificus* differs from *Caenorhabditis elegans* (a bacterial feeder) by its buccal teeth and the lack of pharyngeal grinders, but almost nothing is known about which genes coordinate *P. pacificus* feeding behaviors, such as pharyngeal pumping rate, locomotion, and fat storage.

## Methodology/principal findings

We analyzed *P. pacificus* pharyngeal pumping rate and locomotion behavior on and off food, as well as on different species of bacteria (*Escherichia coli. Bacillus subtilis*, and *Caulobacter crescentus*)*.* We found that the cGMP-dependent protein kinase G (PKG) Ppa-EGL-4 in *P. pacificus* plays an important role in regulating the pumping rate, mouth form dimorphism, the duration of forward locomotion, and the amount of fat stored in intestine. In addition, Ppa-EGL-4 interacts with Ppa-OBI-1, a recently identified protein involved in chemosensation, to influence feeding and locomotion behavior. We also found that *C. crescentus* NA1000 increased pharyngeal pumping as well as fat storage in *P. pacificus.*

## Conclusions

The PKG EGL-4 has conserved functions in regulating feeding behavior in both *C. elegans* and *P. pacificus* nematodes. The Ppa-EGL-4 also has been co-opted during evolution to regulate *P. pacificus* mouth form dimorphism that indirectly affect pharyngeal pumping rate. Specifically, the lack of Ppa-EGL-4 function increases pharyngeal pumping, time spent in forward locomotion, and fat storage, in part as a result of higher food intake. Ppa-OBI-1 functions upstream or parallel to Ppa-EGL-4. The beetle-associated omnivorous *P. pacificus* respond differently to changes in food state and food quality compared to the exclusively bacteriovorous *C. elegans.*

